# Real‐World Diagnostic Performance of Repeat Transthoracic Echocardiography Within a Multimodality Imaging Approach in Patients With Suspected Infective Endocarditis

**DOI:** 10.1161/JAHA.126.050020

**Published:** 2026-08-07

**Authors:** Georgios Tzimas, Pierre Monney, Panagiotis Antiochos, Denise Auberson, Nicoleta Ianculescu, Benoit Guery, Lars Niclauss, Matthias Kirsch, Matthaios Papadimitriou‐Olivgeris

**Affiliations:** ^1^ Department of Cardiology Lausanne University Hospital and University of Lausanne Lausanne Switzerland; ^2^ Infectious Diseases Service Lausanne University Hospital and University of Lausanne Lausanne Switzerland; ^3^ Department of Cardiac Surgery Lausanne University Hospital and University of Lausanne Lausanne Switzerland; ^4^ Infectious Diseases Service Hospital of Valais and Institut Central des Hôpitaux Sion Switzerland

**Keywords:** [18F]FDG‐PET/CT, European Society of Cardiology, infective endocarditis, transesophageal echocardiography, transthoracic echocardiography, Echocardiography

## Abstract

**Background:**

Echocardiography is central to the diagnosis of infective endocarditis (IE). Although guidelines recommend repeating echocardiography when clinical suspicion persists despite negative or inconclusive findings, the diagnostic value of repeating transthoracic echocardiography (TTE) remains uncertain. The aim was to evaluate the diagnostic performance of repeat TTE in the diagnosis of IE among patients with suspected IE.

**Methods:**

This retrospective study conducted at Lausanne University Hospital (2015–2024) including adult patients with suspected IE who underwent 2 TTEs within 21 days. IE was classified according to the 2023 Duke‐International Society for Cardiovascular Infectious Diseases criteria.

**Results:**

A total of 503 episodes were included, of which 254 (50%) were diagnosed as IE. The second TTE was performed within 8 days from first TTE (interquartile range, 5–13 days). The second TTE identified new vegetations in 34 (7%) episodes and new paravalvular complications in 17 (3%) episodes. Sensitivity for detecting vegetations increased from 47% (95% CI, 39%–56%) for the first TTE to 57% (95% CI, 48%–65%) for the second. By excluding the results of cardiac imaging modalities other than TTE, the sensitivity of the second TTE for diagnosing IE was higher (45% [95% CI, 38%–51%]), compared with 34% (95% CI, 28%–41%) for the first TTE; the second TTE led to reclassification from possible to definite IE in 7 (1%) of cases.

**Conclusions:**

Repeat TTE modestly improved typical IE lesion detection; however, within a multimodality imaging strategy, its incremental diagnostic contribution was limited. These results suggest that repeated TTE alone is unlikely to substantially alter diagnosis in most patients, supporting the use of complementary imaging when suspicion of IE remains high.

**Nonstandard Abbreviations and Acronyms**
[^18^F]FDG‐PET/CT: [^18^F]fluorodeoxyglucose positron emission tomography/computed tomography
IE: infective endocarditis
ISCVID: International Society for Cardiovascular Infectious Diseases
TEE: transesophageal echocardiography
TTE: transthoracic echocardiography

**Clinical Perspective**

**What Is New?**

- This study provides a comprehensive real‐world evaluation of the incremental diagnostic value of repeat transthoracic echocardiography within a contemporary multimodality imaging pathway for suspected infective endocarditis.
- Repeat transthoracic echocardiography modestly increased the detection of vegetations and other typical IE lesions but reclassified only 1% of episodes from possible to definite infective endocarditis according to the 2023 Duke‐International Society for Cardiovascular Infectious Diseases clinical criteria.

**What Are the Clinical Implications?**

- When clinical suspicion of infective endocarditis persists despite negative or inconclusive initial echocardiography, higher‐sensitivity imaging modalities, particularly transesophageal echocardiography and, when appropriate, cardiac computed tomography or [18F]fluorodeoxyglucose positron emission tomography/computed tomography, should be prioritized over repeat transthoracic echocardiography, with imaging findings interpreted alongside clinical and microbiological data within a multidisciplinary endocarditis team.

Infective endocarditis (IE) remains a major diagnostic challenge due to its heterogeneous clinical presentation and the variable sensitivity of available diagnostic tools.^1^, ^2^ Echocardiography plays a central role in the diagnostic workup, providing key imaging evidence to support the clinical diagnosis of IE.^1^, ^2^ Moreover, echocardiographic abnormalities constitute a major criterion in the Duke diagnostic frameworks, underscoring its pivotal role in confirming or excluding the diagnosis of IE.^1^, ^3^

Transthoracic echocardiography (TTE) is recommended as the first‐line imaging modality because it is noninvasive, widely available, and provides rapid assessment of cardiac structures. However, its sensitivity for detecting vegetations is limited, typically ranging between 40% and 63%, depending on vegetation size and patient characteristics.^4^ Consequently, when TTE is negative but clinical suspicion remains high, transesophageal echocardiography (TEE) is recommended. Owing to its close proximity to cardiac structures and the use of higher‐frequency transducers, TEE provides superior spatial resolution and overall image quality, resulting in greater sensitivity for detecting vegetations or paravalvular complications.^1^, ^2^

Despite its diagnostic superiority, TEE can yield false‐negative results, particularly in early disease, when vegetations are small, or before the development of detectable perivalvular complications that may evolve over time. When TEE findings remain negative or inconclusive but clinical suspicion persists, complementary imaging modalities should be considered. Cardiac computed tomography (CT) is useful in both native and prosthetic valve IE, particularly for identifying perivalvular complications such as abscesses or pseudoaneurysms, for which it may outperform TEE.^1^, ^2^, ^5^, ^6^, ^7^ In contrast, [^18^F]fluorodeoxyglucose positron emission tomography/CT ([^18^F]FDG‐PET/CT) is mainly recommended in suspected prosthetic valve or cardiac implantable electronic device‐related IE when echocardiographic findings are inconclusive.^1^, ^2^, ^5^, ^8^

An additional diagnostic approach in patients with persistently high clinical suspicion of IE despite negative initial imaging is repeat echocardiography.^1^, ^2^ The rationale for repeating echocardiography lies in the dynamic nature of IE: vegetations can increase in size and become detectable over time or new valvular or perivalvular complications such as leaflet perforation, abscesses, or fistulas may emerge.^9^, ^10^, ^11^ Current guidelines differ regarding the recommended modality for repeat echocardiography when clinical suspicion remains high despite negative or inconclusive initial imaging. The American Heart Association advises repeating TEE after 7 to 10 days, whereas the European Society of Cardiology recommends repeating either TTE or TEE after 5 to 7 days.^1^, ^2^

This study aims to evaluate the diagnostic performance and clinical contribution of repeat TTE for the diagnosis of infective endocarditis within contemporary multimodality imaging pathways among patients with suspected IE.

## METHODS

The data that support the findings of this study are available from the corresponding author on reasonable request.

### Study Design and Population

This retrospective study was conducted at Lausanne University Hospital and covered the period from January 2015 to December 2024. Cases were identified from 2 institutional cohorts: (1) a cohort of patients with suspected IE spanning from January 2015 to December 2024, and (2) a cohort of patients with bacteremia or candidemia enrolled between January 2015 and December 2021. Suspected IE was defined as the combination of blood cultures being drawn and echocardiography performed specifically to investigate IE. The study was approved by the Ethics Committee of the Canton of Vaud, Switzerland (CER‐VD 2021‐02516 and CER‐VD 2017‐02137). Owing to the retrospective nature of the study, the requirement for informed consent was waived by the Ethics Committee; however, patients who had previously refused the use of their data for research purposes were excluded.

Inclusion criteria were adult patients (≥18 years) with suspected IE; performance of a second TTE within 21 days of the initial TTE; and the absence of patient refusal of data use. Duplicate episodes identified across the 2 cohorts, as well as those in which the second TTE was performed after valve surgery or cardiac implantable electronic device extraction, were excluded.

### Definitions and Classification

We manually collected demographic, clinical, imaging, and microbiological information from patients' health records. Infectious diseases consultation was mandatory for all patients with suspected IE. Episodes were diagnosed as IE or no‐IE by the endocarditis team (from January 2018 to June 2024) or by expert clinicians (2015–2017; M.P.O., P.M.).^12^ Episodes were further classified as rejected, possible, or definite IE using the 2023 Duke‐International Society for Cardiovascular Infectious Diseases clinical criteria.^3^ We applied 3 sets of classifications: (1) using imaging findings from cardiac imaging studies performed before the second TTE, (2) including the results of the second TTE, and (3) reflecting the final classification after all cardiac imaging studies had been completed. In episodes with vegetation identified in both TTE, a significant increase or decrease of the vegetation size was considered if the change was >3 mm between 2 TTEs.

### Echocardiography Protocol

When a treating physician requests an echocardiographic examination, they must specify whether it is a standard study or one specifically aimed at investigating IE. In the latter case, the examination is performed or reviewed by senior cardiologists (G.T., P.M. with expertise in IE imaging. In our center, TTE image quality is classified by the performing cardiologist as poor, mediocre, adequate, or good/excellent. The designation of good/excellent quality is strictly reserved for examinations in which all cardiac structures can be assessed in detail and all myocardial segments are adequately visualized. For the purposes of this study, only examinations rated as good/excellent were considered of good quality. TTE was considered positive if it demonstrated the presence of a vegetation, abscess, leaflet perforation, prosthetic valve dehiscence, aneurysm, pseudoaneurysm, or fistula. TTE examinations were performed using Philips EPIQ systems with S5‐1 and X5‐1 transducers, Philips Affiniti systems with S5‐1 transducers, GE Vivid S70 systems with M5Sc‐D transducers, and GE Vivid E9 systems with M5Sc‐D transducers, according to routine clinical practice.

### Statistical Analysis

We used SPSS version 26.0 (SPSS, Chicago, IL, USA) for the statistical analyses. After checking for normality using the Shapiro–Wilk test, continuous variables were assessed by either Mann–Whitney *U* or Welch test as appropriate. Categorical variables were compared using the chi‐square or Fisher's exact test as appropriate. The diagnostic performance of the first and second TTE for detecting specific lesions, namely vegetation, abscess, aneurysm, pseudoaneurysm, fistula, leaflet perforation, and prosthetic valve dehiscence, was evaluated among episodes with suspected IE in which a reference standard (TEE, cardiac CT, [^18^F]FDG‐PET/CT, valve surgery, or autopsy) was available. By excluding the results of cardiac imaging modalities other than TTE, the diagnostic performance of the first and second TTE for diagnosing IE was further evaluated by assessing the concordance between lesions detected on TTE and the final IE diagnosis according to the reference standard. We calculated sensitivity, specificity, positive and negative predictive values, positive and negative likelihood ratios, and accuracy with the corresponding 95% CI using exact binomial methods. Accuracy was defined as the proportion of correctly classified episodes among all evaluated episodes. Analyses were performed at the episode level and assumed independence between episodes, as recurrent episodes from the same patient were infrequent and represented distinct clinical evaluations separated in time. No data were missing in the included variables. All tests were 2 tailed, and *P*<0.05 was considered statistically significant.

## RESULTS

A total of 503 episodes in 486 patients were included from both cohorts, comprising 339 episodes from the cohort of suspected IE and 164 nonduplicate episodes from the cohort of bacteremia/candidemia (Figure 1). Seventeen episodes represented recurrent episodes in the same patient, with a median interval of 197 days between episodes (range, 63–2423 days). The first TTE was performed at a median of 2 days after blood culture collection (interquartile range, 1–4 days), and the second TTE at a median of 8 days after the first examination (interquartile range, 5–13 days). In 347 (69%) episodes, TTE was repeated due to persistent clinical suspicion of IE, as no typical IE lesions had been identified on prior imaging. In the remaining 156 (31%) episodes, in which typical IE lesions had already been detected by prior cardiac imaging, repeat TTE was performed for assessment of complications or follow‐up. Among the initial TTEs, 64 (13%) were considered of good quality, compared with 80 (16%) of the second TTEs. Overall, 284 (56%) episodes underwent TEE, 137 (27%) [^18^F]FDG‐PET/CT, and 37 (7%) cardiac CT. In addition, 94 (19%) episodes required valve surgery and 9 (2%) underwent autopsy. In total, 343 (68%) episodes had at least 1 reference investigation (TEE, [^18^F]FDG‐PET/CT, cardiac CT, valve surgery, or autopsy); among them, 244 episodes had the second TTE within 10 days from the first. The timing of the second TTE relative to other cardiac imaging examinations (TEE, [^18^F]FDG‐PET/CT, cardiac CT) is summarized in Table S1–S5. Of the 503 episodes, 262 (52%) episodes had no other cardiac imaging modalities (TEE, [^18^F]FDG‐PET/CT, cardiac CT) before the second TTE, and among these, 87 (33%) episodes underwent additional cardiac imaging after the second TTE.

**Figure 1:**
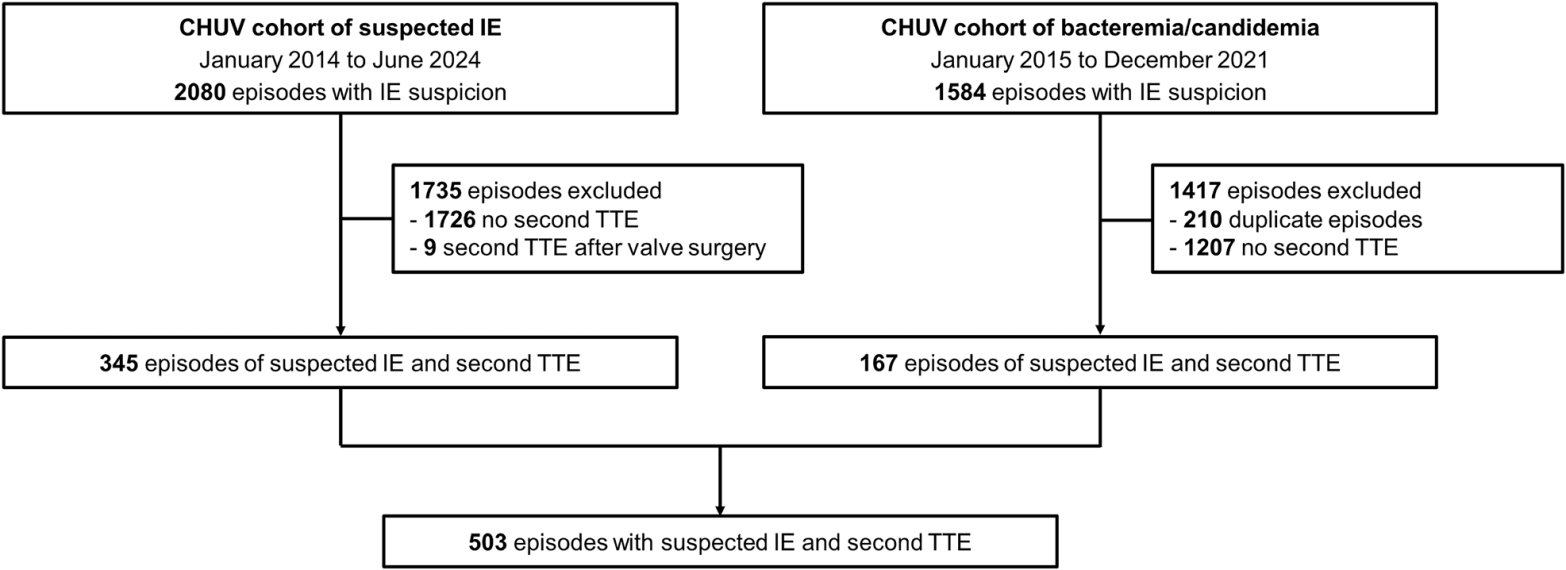
Flow chart of included patients from the 2 cohorts. CHUV indicates Lausanne University Hospital; IE, infective endocarditis; and TTE, transthoracic echocardiography.

IE was diagnosed by the endocarditis team or expert clinicians in 254 (50%) episodes, involving 161 (63%) native valves, 85 (33%) prosthetic valves, and 24 (9%) cardiac implantable electronic device leads. On the first TTE, vegetations were identified in 94 (19%) episodes, abscesses in 2 (0.4%), leaflet perforation or prosthetic valve dehiscence in 2 (0.4%), and significant new valvular regurgitation in 26 (5%) (Table 1). On the second TTE, vegetations were detected in 112 (22%) episodes, abscesses in 10 (2%), leaflet perforation or prosthetic valve dehiscence in 9 (2%), aneurysm, pseudoaneurysm, or fistula in 4 (0.8%), and significant new valvular regurgitation in 48 (10%).

**Table 1:** Comparison of Episodes With and Without IE Diagnosis Among 503 Episodes With Suspected IE Who Underwent a Second TTE

	No infective endocarditis (N=249)	Infective endocarditis (N=254)	*P* value	OR (95% CI)
Demographics				
Male sex, n (%)	172 (69)	183 (72)	0.556	1.14 (0.78–1.67)
Age, y, median (IQR)	66 (56–76)	67 (50–76)	0.370	…
Cardiac predisposing factors				
Intravenous drug use, n (%)	12 (5)	26 (10)	0.027	2.25 (1.10–4.57)
Prior endocarditis, n (%)	9 (4)	27 (11)	0.003	3.17 (1.46–6.89)
Congenital disease, n (%)	12 (5)	27 (11)	0.019	2.35 (1.16–4.75)
Moderate or severe valve regurgitation/stenosis, n (%)	34 (14)	25 (10)	0.213	0.69 (0.40–1.20)
Surgical prosthetic valve or transcatheter aortic valve implantation, n (%)	18 (7)	95 (37)	<0.001	7.67 (4.46–13.2)
CIED, n (%)	24 (10)	42 (17)	0.025	1.86 (1.09–3.17)
Microbiological data				
Bacteremia/candidemia, n (%)	212 (85)	239 (94)	0.001	2.78 (1.48–5.21)
*Staphylococcus aureus*, n (%)	119 (48)	120 (47)	0.929	0.98 (0.69–1.39)
Coagulase‐negative staphylococci, n (%)	20 (8)	13 (5)	0.210	0.62 (0.30–1.27)
*Streptococcus* spp., n (%)	40 (16)	63 (25)	0.020	1.72 (1.11–2.68)
*Enterococcus* spp., n (%)	21 (8)	25 (10)	0.644	1.19 (0.65–2.18)
Gram‐positive (other than staphylococci, streptococci, and enterococci), n (%)	5 (2)	4 (2)	0.750	0.78 (0.21–2.94)
HACEK, n (%)	2 (0.8)	6 (2)	0.285	2.99 (0.60–14.9)
Gram‐negative (other than HACEK), n (%)	20 (8)	11 (4)	0.097	0.52 (0.24–1.11)
*Candida* spp., n (%)	13 (5)	6 (2)	0.106	0.44 (0.16–1.17)
Positive serology for *C. burnetii* or *Bartonella* spp., n (%)	2 (0.8)	0 (0)	0.245	1.71 (0.31–9.5)
Setting of infection onset			<0.001	
Community, n (%)	102 (41)	189 (74)		Reference
Health care‐associated, n (%)	39 (16)	33 (13)		0.46 (0.27–0.77)
Nosocomial, n (%)	108 (43)	32 (13)		0.16 (0.10–0.25)
Cardiac imaging				
Time elapsed between first and second TTE, d, median (IQR)	8 (5–13)	7 (4–12)	0.370	…
TEE, n (%)	95 (38)	189 (74)	<0.001	4.71 (3.22–6.90)
Typical lesion* of IE found on TEE, n (%)	4 (2)	130 (51)	<0.001	64.2 (23.2–178)
Cardiac CT, n (%)	6 (2)	31 (12)	<0.001	5.66 (2.32–13.8)
Typical lesion* of IE found on cardiac CT, n (%)	0 (0)	27 (11)	<0.001	…
[^18^F]FDG‐PET/CT, n (%)	56 (23)	81 (32)	0.021	1.61 (1.08–2.40)
Cardiac abnormal metabolic activity on [^18^F]FDG‐PET/CT, n (%)	1 (0.4)	51 (20)	<0.001	62.3 (8.54–455)
Manifestations				
Fever (>38 °C), n (%)	201 (81)	221 (87)	0.068	1.60 (0.99–2.59)
Sepsis or septic shock, n (%)	113 (45)	116 (46)	1.000	1.01 (0.71–1.44)
Embolic events, n (%)	42 (17)	162 (64)	<0.001	8.68 (5.71–13.2)
Native bone and joint infection, n (%)	50 (20)	48 (19)	0.822	0.99 (0.57–1.37)
Immunologic phenomena, n (%)	6 (2)	28 (11)	<0.001	5.02 (2.04–12.3)
Management of IE				
Valve surgery performed, n (%)	3 (1)	92 (36)	<0.001	46.6 (14.5–150)
CIED‐extraction (among 66 patients with CIED), n (%)	3 (13)	21 (50)	0.005	7.00 (1.81–27.1)
Autopsy performed, n (%)	2 (0.8)	7 (3)	0.176	3.90 (0.78–19.5)
Histopathology (surgery/autopsy) compatible for IE, n (%)	0 (0)	38 (15)	<0.001	…
Positive culture of vegetation, abscess, n (%)	0 (0)	35 (14)	<0.001	…
Positive nucleic acid‐based test of vegetation, abscess, n (%)	0 (0)	16 (6)	<0.001	…
Macroscopic evidence of IE by inspection (surgery/autopsy), n (%)	0 (0)	82 (32)	<0.001	…
Types of cardiac lesion found on TTE, TEE, [^18^F]FDG‐PET/CT, cardiac CT, valve surgery, or autopsy				
Vegetation, n (%)	7 (3)	169 (67)	<0.001	68.7 (31.0–152)
Abscess, n (%)	0 (0)	55 (22)	<0.001	…
Leaflet perforation, dehiscence of prothesis, n (%)	0 (0)	34 (13)	<0.001	…
Aneurysm, pseudoaneurysm, fistula, n (%)	0 (0)	15 (6)	<0.001	…
Leaflet thickening, n (%)	12 (5)	54 (21)	<0.001	5.33 (2.78–10.2)
Significant new valvular regurgitation, n (%)	8 (3)	53 (21)	<0.001	7.94 (3.69–17.1)
Types of cardiac lesions found on first TTE				
Vegetation, n (%)	5 (2)	89 (35)	<0.001	26.3 (10.5–66.2)
Abscess, n (%)	0 (0)	2 (0.8)	0.499	…
Leaflet perforation, dehiscence of prothesis, n (%)	0 (0)	2 (0.8)	0.499	…
Aneurysm, pseudoaneurysm, fistula, n (%)	0 (0)	0 (0)	…	…
Leaflet thickening, n (%)	8 (3)	23 (9)	0.009	3.00 (1.32–6.84)
Significant new valvular regurgitation, n (%)	5 (2)	21 (8)	0.002	4.40 (1.63–11.9)
Types of cardiac lesions found on second TTE				
Vegetation, n (%)	6 (2)	106 (42)	0.002	29.0 (12.4–67.7)
Abscess, n (%)	0 (0)	10 (4)	0.002	…
Leaflet perforation, dehiscence of prothesis, n (%)	0 (0)	9 (4)	0.004	…
Aneurysm, pseudoaneurysm, fistula, n (%)	0 (0)	4 (2)	0.124	…
Leaflet thickening, n (%)	5 (2)	26 (10)	<0.001	5.33 (2.01–14.1)
Significant new valvular regurgitation, n (%)	4 (2)	44 (17)	<0.001	12.8 (4.54–36.3)
New lesions found on second TTE compared with first one				
Vegetation, n (%)	2 (0.8)	32 (13)	<0.001	17.8 (4.22–75.1)
Abscess, leaflet perforation, dehiscence of prothesis, aneurysm, pseudoaneurysm, fistula, n (%)	0 (0)	17 (7)	<0.001	…
Typical IE lesion in another valve, n (%)	0 (0)	3 (1)	0.249	…
Significant new valvular regurgitation, n (%)	2 (0.8)	25 (10)	<0.001	13.5 (3.16–57.6)
Change in vegetation size (among 78 episodes with vegetation in both TEE)			0.672	
Increase by >3 mm, n (%)	0 (0)	16 (22)		…
Similar (−3 mm to 3 mm), n (%)	4 (100)	53 (72)		…
Decrease by >3 mm, n (%)	0 (0)	5 (7)		…
Major imaging criterion from cardiac imaging studies performed before the second TTE, n (%)	13 (5)	152 (59)	<0.001	27.1 (14.7–49.9)
Major imaging criterion including the results of the second TTE, n (%)	15 (6)	166 (64)	<0.001	29.4 (16.4–52.7)
Major imaging criterion after all cardiac imaging studies had been completed, n (%)	16 (6)	208 (80)	<0.001	59.4 (32.8–108)
Classification according to ISCVID criteria without findings from second TTE			<0.001	
Rejected, n (%)	101 (41)	4 (2)		Reference
Possible, n (%)	141 (57)	62 (24)		11.1 (3.91–31.5)
Definite, n (%)	7 (3)	188 (74)		678 (194–2372)
Classification according to ISCVID criteria with findings from second TTE			<0.001	
Rejected, n (%)	97 (39)	4 (2)		Reference
Possible, n (%)	144 (58)	56 (22)		9.43 (3.31–26.9)
Definite, n (%)	8 (3)	194 (76)		588 (173–2001)

[^18^F]FDG‐PET/CT indicates [^18^F]fluorodeoxyglucose positron emission tomography/computed tomography; CIED, cardiac implantable electronic device; CT, tomography; HACEK, Haemophilus, Aggregatibacter, Cardiobacterium, Eikenella, and Kingella; IE, infective endocarditis; IQR, interquartile range; ISCVID, International Society for Cardiovascular Infectious Diseases; OR, odds ratio; TEE, transesophageal echocardiography; and TTE, transthoracic echocardiography.

^*^ Vegetation, leaflet perforation, dehiscence of prosthesis, abscess, aneurysm, pseudoaneurysm, fistula.

Compared with the first TTE, the second TTE newly identified 34 (7%) vegetations, 17 (3%) abscesses, leaflet perforations, prosthetic dehiscence, aneurysms, pseudoaneurysms, or fistulas, 27 (5%) cases of new significant valvular regurgitation, and 3 (0.6%) episodes with new typical lesions on a second valve (Figure 2). Among the 78 episodes in which vegetations were visualized on both TTEs, a significant increase in vegetation size (>3 mm) was observed in 16 (21%) cases, a significant decrease (>3 mm) in 5 (6%), and no significant change was noted in 57 (73%). Among the 94 episodes in which vegetations were visualized on the first TTE, they were no longer observed on the second examination in 16 (17%) episodes. Table S2 presents the comparison between episodes with a positive and negative second TTE among the 220 IE episodes for which at least 1 reference investigation (TEE, [^18^F]FDG‐PET/CT, cardiac CT, valve surgery, or autopsy) was available.

**Figure 2:**
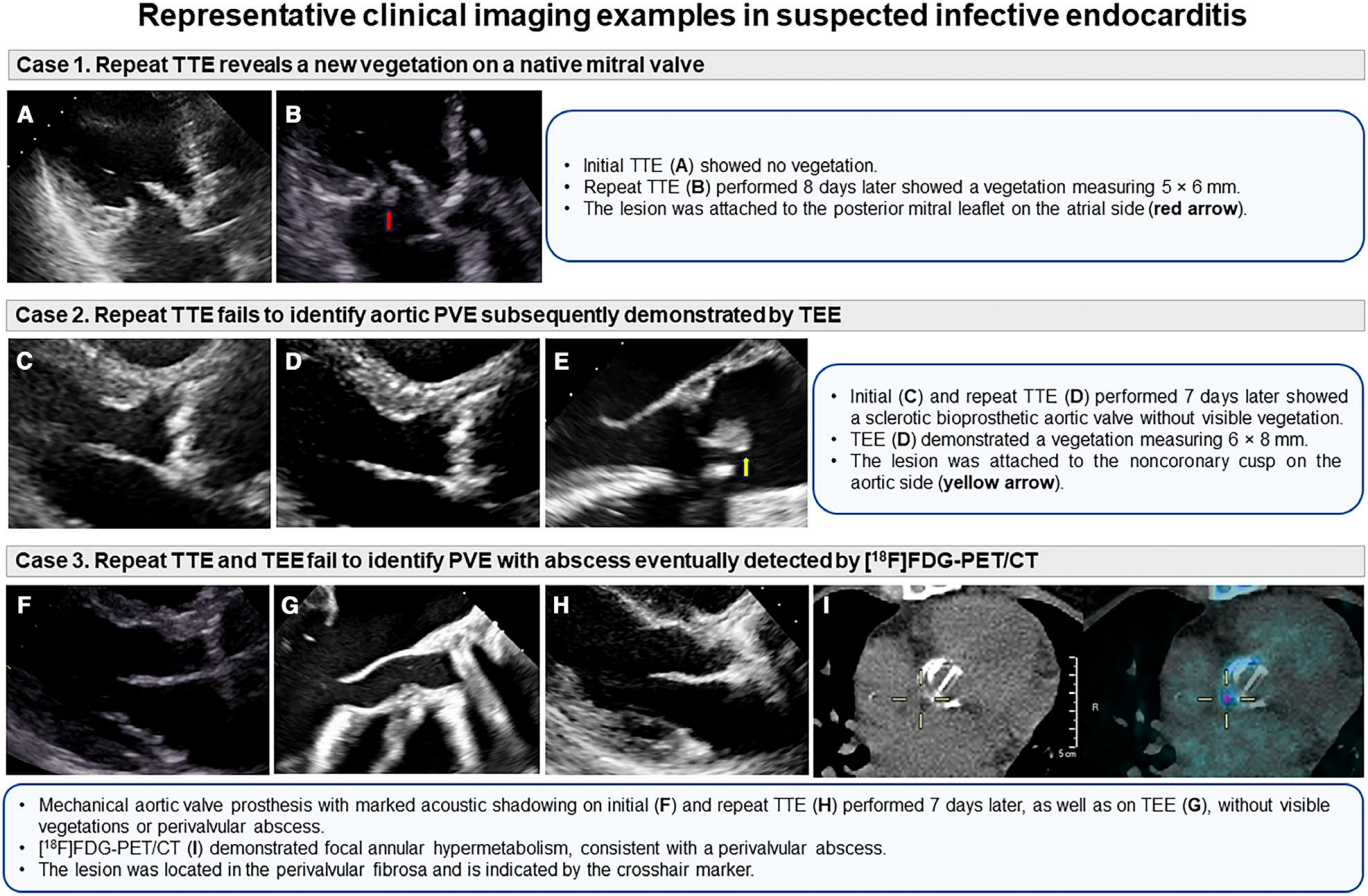
Representative clinical cases illustrating the incremental value of repeat TTE and multimodality imaging in suspected infective endocarditis. [^18^F]FDG‐PET/CT indicates [^18^F]fluorodeoxyglucose positron emission tomography/CT; PVE, prosthetic valve endocarditis; TEE, transesophageal echocardiography; and TTE, transthoracic echocardiography.

By evaluating the cardiac imaging examinations performed before the second TTE, 165 (32%) episodes fulfilled a major imaging criterion according to the 2023 Duke‐International Society for Cardiovascular Infectious Diseases classification. When the results of the second TTE were included, an additional 16 (3%) episodes met this criterion, for a total of 181 (35%) episodes. After incorporating cardiac imaging examinations performed following the second TTE, 224 (45%) episodes fulfilled the major imaging criterion. Overall, 7 (1%) episodes were reclassified from possible to definite IE based on the findings of the second TTE, and 4 from rejected to possible (0.8%) (Figure 3).

**Figure 3:**
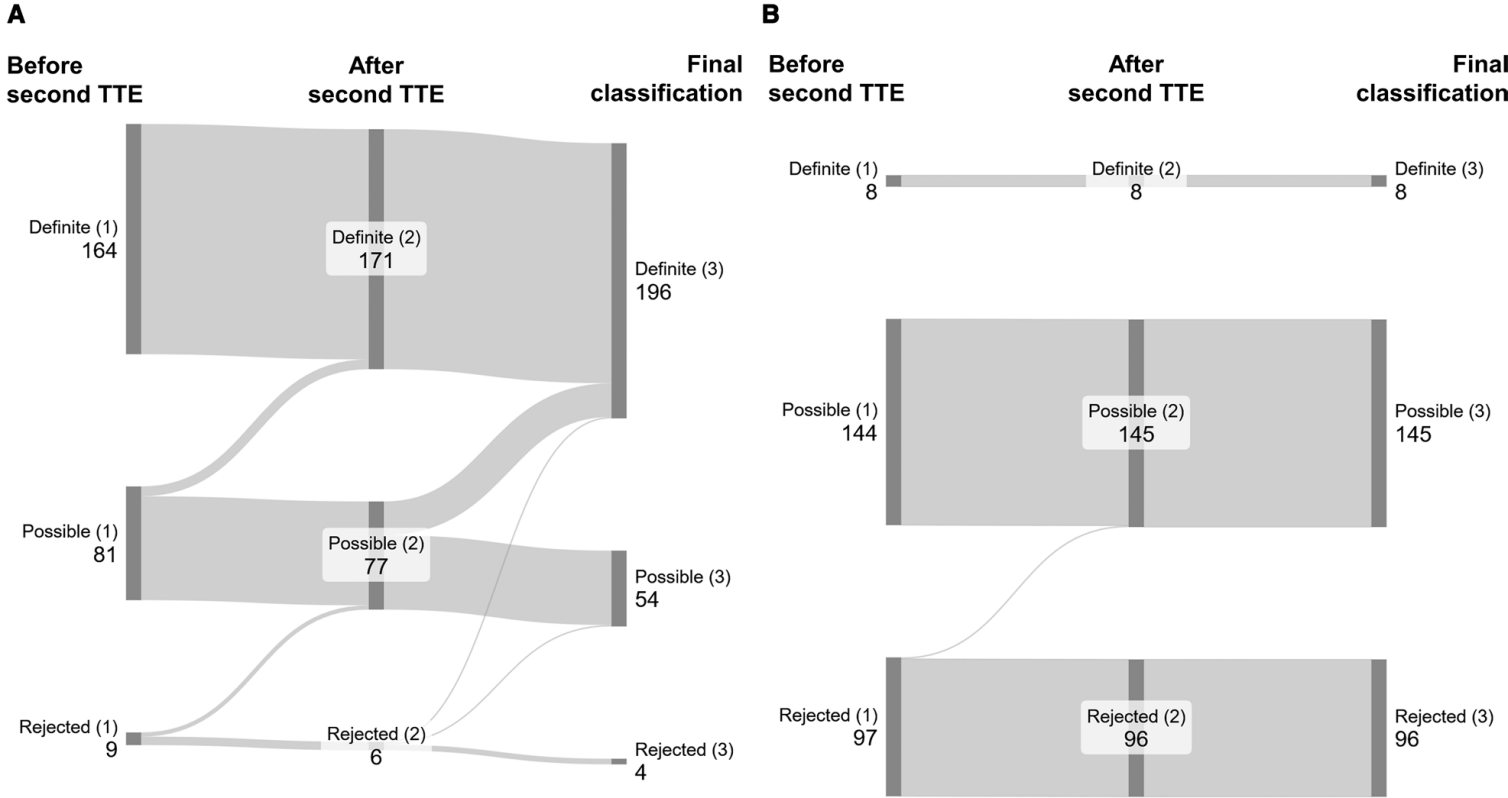
Sankey diagram illustrating changes in infective endocarditis classifications based on the 2023 Duke‐ISCVID clinical criteria. The figure shows episodes (**A**) with infective endocarditis and (**B**) without infective endocarditis across 3 sequential classification sets: (1) using imaging findings from cardiac studies performed before the second TTE; (2) including the results of the second TTE; and (3) reflecting the final classification after all cardiac imaging studies. The diagram highlights reclassifications from rejected to possible infective endocarditis and from possible to definite infective endocarditis over the course of sequential imaging assessments. ISCVID indicates International Society for Cardiovascular Infectious Diseases; and TTE, transthoracic echocardiography.

Table 2 presents the comparison between lesions detected by the first and second TTE and those identified by reference investigations (TEE, [^18^F]FDG‐PET/CT, cardiac CT, valve surgery, or autopsy) among the 343 episodes with suspected IE in which at least 1 of these reference methods was available. The diagnostic performance of the first and second TTE for detecting individual lesions and for the overall diagnosis of IE is summarized in Table 3. The sensitivity for detecting vegetations was 47% (95% CI, 39%–56%) for the first TTE and 57% (95% CI, 48%–65%) for the second TTE. The sensitivity for detecting other typical lesions (abscess, perforation, prosthetic valve dehiscence, aneurysm, pseudoaneurysm, or fistula) increased from 6% (95% CI, 1%–15%) with the first TTE to 22% (95% CI, 13%–34%) with the second. By excluding the results of cardiac imaging modalities other than TTE, the overall sensitivity for diagnosing IE increased from 34% (95% CI, 28%–41%) with the first TTE to 45% (95% CI, 38%–51%) with the second. Table S3 presents the comparison between lesions detected by the first and second TTE (performed within 10 days from the first one) and those identified by reference investigations among the 244 episodes with suspected IE in which at least 1 of these reference methods was available. Table S4 summarizes the diagnostic performance of the first and second TTE among the aforementioned 244 episodes. The results were consistent with the overall analysis, irrespective of the time interval between the first and second TTE. The diagnostic performance of the first and second TTE for the diagnosis of IE was also evaluated in the entire cohort of episodes with suspected IE, regardless of whether reference investigations were performed, and was comparable to the analysis restricted to episodes with at least one reference investigation (Table S5).

**Table 2:** Comparison of Lesions Detected by the First and Second TTE With Corresponding Findings From TEE, [^18^F]FDG‐PET/CT, Cardiac CT, Valve Surgery, or Autopsy Among 343 Episodes With Suspected Infective Endocarditis and at Least 1 Reference Investigation

	TEE, [^18^F]FDG PET/CT, cardiac CT, valve surgery, or autopsy	
	Absence of lesion	Presence of lesion
Vegetation	N=201	No.=142
First TTE, n (%)	8 (4)	67 (47)
Second TTE, n (%)	12 (6)	81 (57)
Abscess, aneurysm, pseudoaneurysm, fistula	N=288	N=55
First TTE, n (%)	0 (0)	2 (4)
Second TTE, n (%)	0 (0)	13 (24)
Leaflet perforation, dehiscence of prothesis	N=313	N=30
First TTE, n (%)	0 (0)	2 (7)
Second TTE, n (%)	3 (1)	5 (17)
Any lesion*	N=186	N=157
First TTE, n (%)	7 (4)	71 (45)
Second TTE, n (%)	10 (5)	93 (59)
Infective endocarditis	N=123	N=220
First TTE, n (%)	3 (2)	75 (34)
Second TTE, n (%)	5 (4)	98 (45)
Infective endocarditis in patients with surgical prosthetic valve or TAVI	N=14	N=81
First TTE, n (%)	1 (7)	13 (16)
Second TTE, n (%)	1 (7)	27 (33)
Infective endocarditis in patients without surgical prosthetic valve or TAVI	N=109	N=139
First TTE, n (%)	2 (2)	62 (45)
Second TTE, n (%)	4 (4)	71 (51)

[^18^F]FDG‐PET/CT indicates [^18^F]fluorodeoxyglucose positron emission tomography/computed tomography; CT, computed tomography; TAVI, transcatheter aortic valve implantation; TEE, transesophageal echocardiography; and TTE, transthoracic echocardiography.

^*^ Vegetation, abscess, leaflet perforation, dehiscence of prothesis, aneurysm, pseudoaneurysm, or fistula.

**Table 3:** Diagnostic Performance of First and Second TTE With Corresponding Findings From TEE, [^18^F]FDG‐PET/CT, Cardiac CT, Valve Surgery, or Autopsy Among 343 Episodes With Suspected IE and at Least One Reference Investigation

	Sensitivity % (95% CI)	Specificity % (95% CI)	PPV % (95% CI)	NPV % (95% CI)	PLR (95% CI)	NLR (95% CI)	Accuracy % (95% CI)
Vegetation							
First TTE	47 (39–56)	96 (92–98)	89 (81–94)	72 (68–75)	11.9 (5.88–23.9)	0.55 (0.47–0.64)	76 (71–80)
Second TTE	57 (48–65)	94 (90–97)	87 (79–92)	76 (74–79)	9.55 (5.42–16.9)	0.46 (0.38–0.55)	79 (74–83)
Abscess, perforation, dehiscence of prothesis, aneurysm, pseudoaneurysm, or fistula							
First TTE	6 (2–15)	100 (99–100)	100 (40–100)	81 (80–82)	‐	0.94 (0.89–1.00)	82 (77–86)
Second TTE	22 (13–34)	99 (97–100)	83 (60–94)	84 (82–86)	20.6 (6.14–69.1)	0.78 (0.69–0.89)	84 (80–88)
Any lesion*							
First TTE	45 (37–54)	96 (92–98)	91 (83–96)	68 (64–71)	12.0 (5.69–25.4)	0.57 (0.49–0.66)	73 (68–76)
Second TTE	59 (51–67)	95 (90–97)	90 (83–95)	73 (69–77)	11.0 (5.95–20.4)	0.43 (036–0.52)	78 (74–83)
Infective endocarditis							
First TTE	34 (28–41)	98 (93–99)	96 (89–99)	45 (43–48)	14.0 (4.50–43.4)	0.68 (0.61–0.75)	57 (51–62)
Second TTE	45 (38–51)	96 (91–99)	95 (89–98)	49 (46–52)	11.0 (4.59–26.2)	0.58 (0.51–0.65)	63 (58–68)
Infective endocarditis among episodes with surgical prosthetic valve or TAVI (n=95 episodes)							
First TTE	17 (8–26)	93 (66–100)	93 (65–99)	16 (14–19)	2.25 (0.32–15.8)	0.90 (0.76–1.08)	27 (19–37)
Second TTE	33 (23–45)	93 (66–100)	96 (77–99)	19 (16–23)	4.67 (0.69–31.6)	0.72 (0.58–0.89)	42 (32–53)
Infective endocarditis among episodes without surgical prosthetic valve or TAVI (n=248 episodes)							
First TTE	45 (36–53)	98 (94–100)	97 (89–99)	58 (54–62)	24.3 (6.08–97.2)	0.56 (0.49–0.66)	68 (62–74)
Second TTE	51 (42–60)	96 (91–99)	95 (87–98)	61 (56–65)	13.9 (5.25–36.9)	0.51 (0.43–0.60)	71 (65–77)

[^18^F]FDG‐PET/CT indicates [^18^F]fluorodeoxyglucose positron emission tomography/ computed tomography; CT, computed tomography; NLR, negative likelihood ratio; NPV, negative predictive value; PLR, positive likelihood ratio; PPV, positive predictive value; TAVI, transcatheter aortic valve implantation; TEE, transesophageal echocardiography; and TTE, transthoracic echocardiography.

^*^ Vegetation, abscess, leaflet perforation, dehiscence of prothesis, aneurysm, pseudoaneurysm, or fistula.

## DISCUSSION

In this study, the second TTE demonstrated better diagnostic performance than the first TTE for detecting typical IE lesions, particularly vegetations. However, within a multimodality imaging strategy, its overall diagnostic performance remained insufficient to safely exclude IE.

The second TTE identified new vegetations in 7% of episodes and new perivalvular complications in 3%. In comparison, previous studies evaluating repeat TEE have reported substantially higher detection rates. Shafiyi et al. found that a second TEE revealed new vegetations and new perivalvular complications in 36% and 8% of cases, respectively.^13^ Similarly, Vieira et al. reported that repeat TTE identified new vegetations and abscesses in 14% and 2%, respectively, whereas repeat TEE detected new vegetations and abscesses in 47% and 35% of cases, respectively.^14^

In our study, the additional diagnostic value of repeat echocardiography was modest, leading to reclassification from possible to definite IE in 1% of patients. This proportion is notably lower than that previously reported for repeat TTE (14%).^14^ This difference is expected, as advanced imaging modalities such as [^18^F]FDG‐PET/CT and cardiac CT were frequently performed in the present study but were not available at the time the study by Vieira et al. was conducted.^14^ Both imaging modalities are now incorporated as major imaging criteria in the 2023 Duke‐International Society for Cardiovascular Infectious Diseases classification and have been shown to significantly improve the diagnostic accuracy for IE.^3^, ^15^ Previous studies evaluating repeat TEE demonstrated markedly higher diagnostic yield, with reclassification rates ranging from 17% to 47%.^13^, ^14^ This difference is expected, given the superior sensitivity of TEE for detecting both vegetations and perivalvular complications.^4^, ^16^ A meta‐analysis reported that, for the detection of vegetations, TTE had a sensitivity of 61% and specificity of 94%, missing many vegetations and most abscesses identified by TEE. The negative likelihood ratios improved when TTE was performed in patients with native valves (negative likelihood ratio=0.36) or when image quality was rated as good (negative likelihood ratio=0.17).^4^ However, in the present study, only a minority of TTEs were considered of good quality, limiting its applicability in clinical practice.

The natural evolution of vegetation size during antimicrobial treatment remains incompletely understood. In the present study, vegetation size remained stable in 72% of cases, increased in 21%, and decreased in 7%. Furthermore, in 17% of episodes in which a vegetation was visualized on the first TTE, it was no longer detected on the second examination. These findings are consistent with previous studies.^9^, ^10^, ^11^ Several investigations have reported that an increase in vegetation size during treatment is associated with a higher risk of embolic events.^10^, ^11^, ^17^ Furthermore, an increase in vegetation size despite appropriate antimicrobial therapy is considered an indication for valve surgery, as it reflects ongoing or uncontrolled infection.^18^ Although the second TTE in our study was performed after a median of 8 days from the initial examination, studies that repeated TTE at the completion of antimicrobial therapy have shown that vegetations often persist in the majority of patients (49%–75%) despite microbiological cure.^9^, ^17^, ^19^ Importantly, the persistence of vegetation after completion of treatment has not been associated with an increased risk of relapse, suggesting that bacterial eradication occurs while the fibrin‐thrombotic scaffold remains.^19^

Overall, these findings suggest that, within contemporary multimodality imaging pathways, repeat TTE contributes limited additional diagnostic value compared with higher‐sensitivity imaging modalities. These results question the European Society of Cardiology recommendation suggesting repetition of either TTE or TEE when clinical suspicion remains high despite negative or inconclusive initial imaging,^18^ and instead support the American Heart Association recommendation to perform repeat TEE, rather than TTE.^2^ However, these recommendations, originally introduced in the first versions of each guideline (American Heart Association 2005, European Society of Cardiology 2003),^20^, ^21^ predate the widespread adoption of advanced imaging modalities such as [^18^F]FDG‐PET/CT and cardiac CT. In current clinical practice, imaging modalities provide complementary diagnostic information. TTE can detect large vegetations, particularly in native valve IE, but may miss small lesions or perivalvular complications.^1^, ^2^, ^4^ TEE improves the detection of small vegetations and abscesses, whereas cardiac CT is particularly useful for assessing perivalvular extension.^1^, ^2^, ^5^, ^6^, ^7^ In addition, [^18^F]FDG‐PET/CT can identify inflammatory activity, especially in prosthetic valve IE.^1^, ^2^, ^5^, ^8^ Particularly in patients with prosthetic valves and inconclusive or negative echocardiography, performing [^18^F]FDG‐PET/CT and/or cardiac CT are often more informative than repeating echocardiography.^1^, ^2^, ^5^, ^6^, ^7^, ^8^ Nevertheless, repeat echocardiography remains a valid option when these modalities are unavailable or contraindicated.^1^, ^2^ Given the limited sensitivity of TTE compared with TEE, repeat TEE should be prioritized whenever feasible.^4^

The present study has several limitations. First, this retrospective single center was performed in an institution with routine access to advanced imaging modalities, including [^18^F]FDG‐PET/CT, and cardiac CT. Consequently, our findings may have limited generalizability to other health care settings with different diagnostic resources.^22^, ^23^ Second, interoperator variability may have influenced echocardiographic findings. However, in our institution, echocardiograms performed to investigate IE are either conducted or reviewed by experienced cardiologists (G.T., P.M.) with specific expertise in IE imaging. Furthermore, since 2018, cases have been systematically reviewed during Endocarditis Team meetings, which likely minimized this effect. Third, the indication for repeating TTE, whether for diagnosis, assessment of complications, or follow‐up, was not systematically recorded in the data set. To provide context, we report the timing of the second TTE relative to other cardiac imaging modalities (TEE, [^18^F]FDG‐PET/CT, cardiac CT) in Tables S1–S5. Overall, 262 (51%) episodes had no other cardiac imaging modalities before the second TTE, and among these, 87 (33%) episodes underwent additional cardiac imaging after the second TTE. Nonetheless, in most cases, the decision to repeat TTE was made during multidisciplinary Endocarditis Team meetings.^12^ Furthermore, because this study reflects real‐world multimodality imaging pathways, the incremental diagnostic value of repeat TTE cannot be fully isolated from the contribution of other imaging modalities. Fourth, selection bias cannot be excluded, as the study population likely included patients with less severe disease; patients who died early or underwent valve surgery soon after the initial evaluation were not included. However, such cases with life‐threatening complications would not typically undergo delayed reassessment with repeat echocardiography, thereby partially mitigating this bias. Fifth, the timing of the repeat TTE varied substantially across episodes. To minimize this potential source of bias, we performed a supplementary analysis restricted to episodes in which the second TTE was conducted within 10 days of the first. This analysis yielded similar findings (Tables S2 and S3). Sixth, the threshold of a >3 mm change in vegetation size to define a significant difference was arbitrary, though it was chosen to minimize the impact of interobserver variability. Finally, in the absence of an absolute reference standard, adjudication relied on endocarditis teams or expert clinicians with full access to clinical, microbiological, imaging, and surgical data. Although some degree of subjectivity is unavoidable, this process reflects best clinical practice and is consistent with current international guideline recommendations advocating for multidisciplinary case adjudication.^1^ However, reliance on the Endocarditis Team's clinical diagnosis as the reference standard introduces potential misclassification bias. To mitigate this, we also applied the 2023 Duke‐International Society for Cardiovascular Infectious Diseases criteria for parallel validation.

## Conclusions

In conclusion, repeat TTE modestly improved the detection of typical IE lesions; however, within a multimodality imaging strategy, its incremental diagnostic contribution was limited with only a small proportion of cases being reclassified. Thus, when available, repeat TEE or advanced imaging modalities such as [^18^F]FDG‐PET/CT or cardiac CT should be preferred when clinical suspicion persists despite negative or inconclusive initial echocardiography. Future studies should directly compare the diagnostic performance of repeat TTE and repeat TEE with that of [^18^F]FDG‐PET/CT and cardiac CT in patients with initially negative or inconclusive echocardiographic findings.

## Sources of Funding

This work was supported by the Swiss National Science Foundation (SNSF) grant #32003B_219351/1 (to Matthaios Papadimitriou‐Olivgeris), the Swiss Heart Foundation grant #FF24013 (to Matthaios Papadimitriou‐Olivgeris, PM), and the Vaudois Foundation for Interventional Cardiology grant (to Matthaios Papadimitriou‐Olivgeris, PM). The funders had no role in study design, data collection and analysis, decision to publish, or preparation of the article.

## Disclosures

None.

## Supporting information

Tables S1–S5

STROBE checklist
