# Supplementary material for: Real‐World Diagnostic Performance of Repeat Transthoracic Echocardiography Within a Multimodality Imaging Approach in Patients With Suspected Infective Endocarditis
Source: J Am Heart Assoc. 2026 Aug 7;15(16):e050020. doi: 10.1161/JAHA.126.050020 (PMC13695718; doi:10.1161/JAHA.126.050020)
Supplement: Supplementary file 2 [file JAH3-15-e050020-s002.pdf]

# Supplemental Material

**Table S1.** Sequence of second TTE relative to other cardiac imaging modalities (TEE, [$^{18}$F]FDG-PET/CT, cardiac CT)

| | **No infective endocarditis (N=252)** | **Infective endocarditis (N=260)** | **All patients (N=512)** |
|---|---|---|---|
| No other cardiac imaging exam performed, n (%) | 131 (52) | 44 (17) | 175 (34) |
| Any cardiac imaging exam performed before second TTE, n (%) | 78 (31) | 172 (66) | 250 (49) |
| Any cardiac imaging exam performed after second TTE, n (%) | 43 (17) | 44 (17) | 87 (17) |

[$^{18}$F]FDG-PET/CT: [$^{18}$F]fluorodeoxyglucose positron emission tomography/CT; TAVI: Transcatheter Aortic Valve Implantation; TTE: transthoracic echocardiography; TEE: transesophageal echocardiography

**Table S2**. Comparison between episodes with a positive and negative second TTE among the 220 IE episodes for which at least one reference investigation (TEE, [$^{18}$F]FDG-PET/CT, cardiac CT, valve surgery, or autopsy) was available.

| | **Negative (n=122)** | **Positive (n=98)** | ***P*** |
|---|---|---|---|
| Demographics | | | |
| Male sex, n (%) | 89 (73) | 70 (71) | 0.880 |
| Age (years), median (IQR) | 67 (55-76) | 59 (40-73) | 0.008 |
| Cardiac predisposing factors | | | |
| Intravenous drug use, n (%) | 5 (4) | 15 (15) | 0.005 |
| Prior endocarditis, n (%) | 14 (12) | 9 (9) | 0.661 |
| Congenital disease, n (%) | 16 (13) | 11 (11) | 0.837 |
| Moderate or severe valve regurgitation/stenosis, n (%) | 14 (12) | 4 (4) | 0.051 |
| Surgical prosthetic valve or TAVI, n (%) | 54 (44) | 27 (28) | 0.012 |
| CIED, n (%) | 23 (19) | 13 (13) | 0.279 |
| Microbiological data | | | |
| Bacteremia/candidemia, n (%) | 117 (96) | 89 (91) | 0.166 |
| Microbiologic cause of IE | | | |
| *S. aureus*, n (%) | 63 (52) | 36 (37) | 0.030 |
| Coagulase-negative staphylococci, n (%) | 4 (3) | 4 (4) | 1.000 |

| | | | |
|---|---|---|---|
| *Streptococcus* spp., n (%) | 27 (22) | 36 (37) | 0.024 |
| *Enterococcus* spp., n (%) | 13 (11) | 7 (7) | 0.481 |
| Gram-positive (other than staphylococci, streptococci and enterococci), n (%) | 2 (2) | 2 (2) | 1.000 |
| HACEK, n (%) | 5 (4) | 1 (1) | 0.229 |
| Gram-negative (other than HACEK), n (%) | 7 (6) | 4 (4) | 0.758 |
| *Candida* spp., n (%) | 0 (0) | 3 (3) | 0.087 |
| Intracellular bacteria, n (%) | 0 (0) | 0 (0) | - |
| Setting of infection onset | | | 0.010 |
| Community, n (%) | 87 (71) | 84 (86) | |
| Healthcare-associated, n (%) | 15 (12) | 10 (10) | |
| Nosocomial, n (%) | 20 (16) | 4 (4) | |
| Cardiac imaging | | | |
| Time elapsed between first and second TTE (days), median (IQR) | 8 (6-13) | 6 (3-10) | 0.008 |
| TEE, n (%) | 108 (89) | 81 (83) | 0.245 |
| Typical lesion[a] of IE found on TEE, n (%) | 55 (45) | 75 (77) | <0.001 |
| Cardiac-CT, n (%) | 15 (12) | 16 (16) | 0.440 |
| Typical lesion[a] of IE found on cardiac CT, n (%) | 13 (11) | 14 (14) | 0.418 |
| [$^{18}$F]FDG-PET/CT, n (%) | 61 (50) | 20 (20) | <0.001 |

| | | | |
|---|---|---|---|
| Cardiac abnormal metabolic activity on [$^{18}$F]FDG-PET/CT, n (%) | 40 (33) | 11 (11) | <0.001 |
| Manifestations | | | |
| Fever (>38ºC), n (%) | 105 (86) | 86 (88) | 0.842 |
| Sepsis or septic shock, n (%) | 55 (45) | 46 (47) | 0.787 |
| Embolic events, n (%) | 69 (57) | 76 (78) | 0.002 |
| Native bone and joint infection, n (%) | 31 (25) | 9 (9) | 0.002 |
| Immunologic phenomena, n (%) | 15 (12) | 12 (12) | 1.000 |
| Type of IE | | | |
| Native valve, n (%) | 65 (53) | 74 (76) | 0.001 |
| Prosthetic valve, n (%) | 50 (41) | 23 (24) | 0.006 |
| CIED lead, n (%) | 17 (14) | 7 (7) | 0.130 |
| Management of IE | | | |
| Valve surgery performed, n (%) | 30 (25) | 62 (64) | <0.001 |
| CIED-extraction (among 36 patients with CIED), n (%) | 12 (52) | 9 (69) | 0.484 |
| Autopsy performed, n (%) | 5 (4) | 2 (2) | 0.465 |
| Histopathology (surgery/autopsy) compatible for IE, n (%) | 13 (11) | 25 (26) | 0.004 |
| Positive culture of vegetation, abscess, n (%) | 9 (7) | 26 (27) | <0.001 |
| Positive nucleic acid-based test of vegetation, abscess s, n (%) | 8 (7) | 8 (8) | 0.795 |
| Macroscopic evidence of IE by inspection (surgery/autopsy), n (%) | 24 (20) | 58 (59) | <0.001 |

| | | | |
|---|---|---|---|
| Types of cardiac lesions found on TTE, TEE, [18F]FDG PET/CT, cardiac CT, valve surgery, or autopsy | | | |
| Vegetation, n (%) | 56 (46) | 95 (97) | <0.001 |
| Abscess, n (%) | 23 (19) | 31 (32) | 0.040 |
| Leaflet perforation, dehiscence of prothesis, n (%) | 9 (7) | 24 (25) | 0.001 |
| Aneurysm, pseudoaneurysm, fistula, n (%) | 8 (7) | 7 (7) | 1.000 |
| Leaflet thickening, n (%) | 30 (25) | 22 (22) | 0.751 |
| Significant new valvular regurgitation, n (%) | 17 (14) | 35 (36) | <0.001 |
| Types of cardiac lesions found on first TTE | | | |
| Vegetation, n (%) | 13 (11) | 59 (60) | <0.001 |
| Abscess, n (%) | 0 (0) | 2 (2) | 0.197 |
| Leaflet perforation, dehiscence of prothesis, n (%) | 0 (0) | 2 (2) | 0.197 |
| Aneurysm, pseudoaneurysm, fistula, n (%) | 0 (0) | 0 (0) | - |
| Leaflet thickening, n (%) | 10 (0) | 12 (12) | <0.001 |
| Significant new valvular regurgitation, n (%) | 8 (7) | 12 (12) | 0.163 |
| Types of cardiac lesions found on second TTE | | | |
| Vegetation, n (%) | 0 (0) | 88 (90) | <0.001 |
| Abscess, n (%) | 0 (0) | 9 (9) | 0.001 |
| Leaflet perforation, dehiscence of prothesis, n (%) | 0 (0) | 8 (8) | 0.001 |
| Aneurysm, pseudoaneurysm, fistula, n (%) | 0 (0) | 4 (4) | 0.038 |

| | | | |
|---|---|---|---|
| Leaflet thickening, n (%) | 13 (11) | 11 (11) | 1.000 |
| Significant new valvular regurgitation, n (%) | 13 (11) | 30 (31) | <0.001 |

[a]Vegetation, leaflet perforation, abscess, aneurysm, pseudoaneurysm, fistula

[$^{18}$F]FDG-PET/CT: [$^{18}$F]fluorodeoxyglucose positron emission tomography/CT; CIED: cardiac implantable electronic device; IE: Infective endocarditis; ISCVID: International Society for Cardiovascular Infectious Diseases; TAVI: Transcatheter Aortic Valve Implantation; TTE: transthoracic echocardiography; TEE: transesophageal echocardiography

**Table S3.** Comparison of lesions detected by the first and second TTE (performed within 10 days from the first one) with corresponding findings from TEE, [18F]FDG-PET/CT, cardiac CT, valve surgery or autopsy among 244 episodes with suspected infective endocarditis and at least one reference investigation

| | **TEE, [18F]FDG PET/CT, cardiac CT, valve surgery, or autopsy** | |
|---|---|---|
| | **Absence of lesion** | **Presence of lesion** |
| **Vegetation** | **N=136** | **N=108** |
| First TTE, n (%) | 7 (5) | 52 (48) |
| Second TTE, n (%) | 9 (7) | 65 (60) |
| **Abscess, aneurysm, pseudoaneurysm, fistula** | **N=200** | **N=42** |
| First TTE, n (%) | 0 (0) | 2 (5) |
| Second TTE, n (%) | 0 (0) | 8 (18) |
| **Leaflet perforation, dehiscence of prothesis** | **N=222** | **N=22** |
| First TTE, n (%) | 0 (0) | 1 (5) |
| Second TTE, n (%) | 2 (0.9) | 3 (14) |
| **Any lesion[a]** | **N=125** | **N=119** |
| First TTE, n (%) | 6 (5) | 55 (46) |
| Second TTE, n (%) | 8 (6) | 72 (61) |
| **Infective endocarditis** | **N=78** | **N=166** |
| First TTE, n (%) | 2 (3) | 59 (36) |

| | | |
|---|---|---|
| Second TTE, n (%) | 3 (4) | 77 (46) |

[a] vegetation, abscess, leaflet perforation, dehiscence of prothesis, aneurysm, pseudoaneurysm, or fistula

[$^{18}$F]FDG-PET/CT: [$^{18}$F]fluorodeoxyglucose positron emission tomography/CT; TTE: transthoracic echocardiography; TEE: transesophageal echocardiography

**Table S4.** Diagnostic performance of first and second TTE (performed within 10 days from the first one) with corresponding findings from TEE, [18F]FDG-PET/CT, cardiac CT, valve surgery, or autopsy among 244 episodes with suspected IE and at least one reference investigation

| | Sensitivity % (95% CI) | Specificity % (95% CI) | PPV % (95% CI) | NPV % (95% CI) | PLR (95% CI) | NLR (95% CI) | Accuracy % (95% CI) |
|---|---|---|---|---|---|---|---|
| **Vegetation** | | | | | | | |
| First TTE | 48 (38-58) | 95 (90-98) | 88 (78-94) | 70 (66-74) | 9.35 (4.43-19.8) | 0.55 (0.45-0.66) | 74 (68-80) |
| Second TTE | 60 (50-69) | 93 (88-97) | 88 (79-93) | 75 (70-79) | 9.09 (4.75-17.4) | 0.43 (0.33-0.53) | 79 (73-84) |
| **Abscess, perforation, dehiscence of prothesis, aneurysm, pseudoaneurysm, or fistula** | | | | | | | |
| First TTE | 6 (1-16) | 100 (98-100) | 100 (40-100) | 79 (78-80) | - | 0.94 (0.88-1.00) | 80 (74-84) |
| Second TTE | 19 (9-32) | 99 (96-100) | 83 (53-96) | 81 (79-83) | 18.0 (4.07-79.7) | 0.82 (0.72-0.93) | 82 (76-86) |
| **Any lesion[a]** | | | | | | | |
| First TTE | 46 (37-56) | 95 (90-98) | 90 (80-95) | 65 (61-69) | 9.63 (4.31-21.5) | 0.56 (0.48-0.67) | 71 (65-77) |
| Second TTE | 61 (51-69) | 94 (88-97) | 90 (82-95) | 71 (66-76) | 9.45 (4.67-18.8) | 0.42 (034-0.53) | 77 (72-83) |
| **Infective endocarditis** | | | | | | | |
| First TTE | 36 (28-43) | 97 (91-100) | 97 (88-99) | 42 (39-44) | 13.9 (3.48-55.3) | 0.66 (0.59-0.74) | 55 (49-62) |
| Second TTE | 46 (39-54) | 96 (89-99) | 96 (89-99) | 46 (42-49) | 12.1 (3.93-37.0) | 0.56 (0.48-0.65) | 62 (56-68) |

[a] vegetation, abscess, leaflet perforation, dehiscence of prothesis, aneurysm, pseudoaneurysm, or fistula

[18F]FDG-PET/CT: [18F]fluorodeoxyglucose positron emission tomography/CT; NLR: negative likelihood ratio; NPV: negative predictive value; PLR: positive likelihood ratio; PPV: positive predictive value; TTE: transthoracic echocardiography; TEE: transesophageal echocardiography

**Table S5.** Diagnostic performance of first and second TTE among 503 episodes with suspected IE

| | Sensitivity % (95% CI) | Specificity % (95% CI) | PPV % (95% CI) | NPV % (95% CI) | PLR (95% CI) | NLR (95% CI) | Accuracy % (95% CI) |
|---|---|---|---|---|---|---|---|
| **Infective endocarditis** | | | | | | | |
| First TTE | 35 (30-42) | 98 (95-99) | 94 (87-98) | 59 (57-62) | 14.9 (6.63-33.2) | 0.66 (0.60-0.73) | 66 (62-70) |
| Second TTE | 45 (38-51) | 97 (94-99) | 94 (89-97) | 63 (60-66) | 16.1 (7.64-33.8) | 0.57 (0.51-0.64) | 71 (66-74) |
| **Infective endocarditis among episodes with surgical prosthetic valve or TAVI (n=117 episodes)** | | | | | | | |
| First TTE | 21 (14-31) | 95 (74-100) | 95 (75-100) | 19 (17-21) | 4.07 (0.58-28.5) | 0.83 (0.72-0.96) | 33 (25-43) |
| Second TTE | 36 (26-46) | 95 (74-100) | 97 (84-100) | 22 (19-26) | 6.79 (0.99-46.6) | 0.68 (0.57-0.81) | 45 (36-55) |
| **Infective endocarditis among episodes without surgical prosthetic valve or TAVI (n=386 episodes)** | | | | | | | |
| First TTE | 44 (36-52) | 98 (95-99) | 93 (85-97) | 71 (69-74) | 20.4 (8.43-49.5) | 0.57 (0.50-0.66) | 76 (71-80) |
| Second TTE | 50 (42-58) | 97 (94-99) | 93 (86-97) | 74 (71-77) | 19.4 (8.68-43.3) | 0.51 (0.44-0.60) | 78 (74-82) |

NLR: negative likelihood ratio; NPV: negative predictive value; PLR: positive likelihood ratio; PPV: positive predictive value; TAVI: Transcatheter Aortic Valve Implantation; TTE: transthoracic echocardiography; TEE: transesophageal echocardiography
